# Prognostic and histogenetic roles of gene alteration and the expression of key potentially actionable targets in salivary duct carcinomas

**DOI:** 10.18632/oncotarget.22927

**Published:** 2017-12-04

**Authors:** Tomotaka Shimura, Yuichiro Tada, Hideaki Hirai, Daisuke Kawakita, Satoshi Kano, Kiyoaki Tsukahara, Akira Shimizu, Soichiro Takase, Yorihisa Imanishi, Hiroyuki Ozawa, Kenji Okami, Yuichiro Sato, Yukiko Sato, Chihiro Fushimi, Hideaki Takahashi, Takuro Okada, Hiroki Sato, Kuninori Otsuka, Yoshihiro Watanabe, Akihiro Sakai, Koji Ebisumoto, Takafumi Togashi, Yushi Ueki, Hisayuki Ota, Mizuo Ando, Shinji Kohsaka, Toyoyuki Hanazawa, Hideaki Chazono, Yoshiyuki Kadokura, Hitome Kobayashi, Toshitaka Nagao

**Affiliations:** ^1^ Department of Otorhinolaryngology, Showa University School of Medicine, Tokyo, Japan; ^2^ Department of Anatomic Pathology, Tokyo Medical University, Tokyo, Japan; ^3^ Department of Head and Neck Oncology and Surgery, International University of Health and Welfare Mita Hospital, Tokyo, Japan; ^4^ Department of Otolaryngology Head and Neck Surgery, Nagoya City University Graduate School of Medical Sciences, Nagoya, Japan; ^5^ Department of Otolaryngology Head and Neck Surgery, Hokkaido University Graduate School of Medicine, Sapporo, Japan; ^6^ Department of Otolaryngology Head and Neck Surgery, Tokyo Medical University, Tokyo, Japan; ^7^ Department of Otolaryngology Head and Neck Surgery, Keio University School of Medicine, Tokyo, Japan; ^8^ Department of Otolaryngology Head and Neck Surgery, Tokai University School of Medicine, Isehara, Japan; ^9^ Department of Head and Neck Surgery, Niigata Cancer Center Hospital, Niigata, Japan; ^10^ Department of Pathology, Cancer Institute Hospital, Japanese Foundation for Cancer Research, Tokyo, Japan; ^11^ Department of Otolaryngology Head and Neck Surgery, Faculty of Medicine, The University of Tokyo, Tokyo, Japan; ^12^ Department of Medical Genomics, Graduate School of Medicine, The University of Tokyo, Tokyo, Japan; ^13^ Department of Otolaryngology, Head and Neck Surgery, Chiba University Graduate School of Medicine, Chiba, Japan; ^14^ Department of Otorhinolaryngology, Showa University Northern Yokohama Hospital, Yokohama, Japan

**Keywords:** salivary duct carcinoma, TP53, PIK3CA, H-RAS, PI3K/Akt signaling pathway

## Abstract

The molecular characteristics of therapeutically-relevant targets and their clinicopathological implications in salivary duct carcinomas (SDCs) are poorly understood. We investigated the gene alterations and the immunoexpression of crucial oncogenic molecules in 151 SDCs. The mutation rates that were identified, in order of frequency, were as follows: *TP53*, 68%; *PIK3CA*, 18%; *H-RAS*, 16%; *BRAF*, 4%; and *AKT1*, 1.5%. *PIK3CA/H-RAS/BRAF* mutations were more common in *de novo* SDC than in SDC ex-pleomorphic adenoma. Furthermore, these mutations were mutually exclusive for HER2 overexpression/amplification. *TP53* mutations were frequently detected in cases with the aberrant p53 expression, and *TP53* missense and truncating mutations were associated with p53-extreme positivity and negativity, respectively. DISH analysis revealed no cases of *EGFR* amplification. The rates of PI3K, p-Akt, and p-mTOR positivity were 34%, 22%, and 66%, respectively; PTEN loss was observed in 47% of the cases. These expressions were correlated according to the signaling axis. Cases with PI3K negativity and PTEN loss appeared to show a lower expression of androgen receptor. In the multivariate analysis, patients with SDC harboring *TP53* truncating mutations showed shorter progression-free survival. Conversely, p-Akt positivity was associated with a favorable outcome. This study might provide information that leads to advances in personized therapy for SDC.

## INTRODUCTION

Salivary duct carcinoma (SDC) is an uncommon entity, and histologically resembles high-grade mammary ductal carcinoma [1]. It occurs as a *de novo* carcinoma (*de novo* SDC) or a carcinoma ex-pleomorphic adenoma (SDC ex-PA) [1]. SDCs are known to be one of the most aggressive types of salivary gland carcinoma, since patients frequently develop distant metastasis, even after curative resection [2, 3]. Surgical resection is the standard therapy for SDC; in many cases, postoperative radiotherapy is also performed [4]. Although there is no established regimen, systemic chemotherapy is often carried out in cases with distant metastasis [4, 5].

In the past decade, there have been remarkable advances in the research on multiple therapeutically-relevant genetic alterations in various cancers, and appropriate targeted therapies have been generated [6, 7]. In SDCs, however, there is little data in relation to personalized therapy, with the exception of therapies targeting HER2 and the androgen receptor (AR); evidence for which has gradually accumulated [4, 5]. On the other hand, recent studies in which limited comprehensive molecular profiling analyses were performed—especially those using next-generation sequencing—have revealed that several genes are altered in SDCs, including *TP53* and genes related to cell signaling by receptor-tyrosine kinases, the PI3K/Akt pathway, and the RAS-MAPK cascade, which play integral roles in tumor growth and the survival of various types of carcinoma [8–17]. These studies have reported that the percentages of genetic alteration of *TP53*, *ERBB2*, *PIK3CA*, and *H-RAS*, are characteristically high in SDC. Although mutations of other key genes, such as *CDKN2A*, *PTEN*, *KIT*, and *APC* also have been described in the literature, those frequencies are low, being ≤ 10% for any of them [8–17]. Due to the rarity of the SDCs, the differences in the genetic mechanisms underlying carcinogenesis according to the histologic origin (i.e. *de novo* SDC or SDC ex-PA) [13, 14, 18] and the relationship between the aberrant expression of molecular markers and the clinical outcome have not been fully explored [8, 9, 14, 18]. However, it is essential to clarify these issues as an initial step in designing effective therapeutic options and—in the future—for selecting SDC patients who are candidates for targeted therapy.

The purpose of the present study, the largest cohort of SDCs to date, was to determine the gene alteration and expression status of selected potentially actionable cancer-related targets, and to evaluate their clinicopathological and prognostic significance in SDC patients.

## RESULTS

### Patient characteristics

The distribution of the patient characteristics is shown in Table 1. The median follow-up period of the survivors was 3.7 years (range, 0.4–18.7 years). The 3-year overall survival (OS) and progression-free survival (PFS) rates of the whole study population were 68.5% (95% confidence interval [CI]: 60.1–75.5) and 34.3% (95% CI: 26.7–42.1), respectively.

**Table 1:** Patient characteristics (*n* = 151)

Variables	*n*	%
Age (years)		
< 65	84	56
≥ 65	67	44
Sex		
Male	127	84
Female	24	16
T classification		
1	13	8
2	39	26
3	30	20
4	69	46
N classification		
0	71	47
1	9	6
2	71	47
M classification		
0	142	94
1	9	6
Primary tumor site		
Parotid gland	117	77
Submandibular gland	30	20
Others	4	3
Histologic origin		
*De novo*	57	38
Ex-pleomorphic adenoma^*^	89	59
Unknown	5	3

^*^Salivary duct carcinomas ex-pleomorphic adenoma (SDCs ex-PA) include 13 intracapsular SDC ex-PA cases, 5 minimally invasive SDC ex-PA cases, and 71 widely invasive SDC ex-PA cases.

### Gene alterations and the immunohistochemical findings

*TP53* mutations were the most frequent genetic mutation among all patients with SDC (68%, 85/125 cases), followed by the *PIK3CA* (18%, 25/137 cases), *H-RAS* (16%, 23/140 cases), *BRAF* (3.7%, 5/136 cases), and *AKT1* (1.5%, 2/133 cases) mutations. No *K-RAS* or *N-RAS* mutations were detected in any of the cases. Twenty-four of the patients showed no genetic mutations among the analyzed genes.

**Figure 1:**
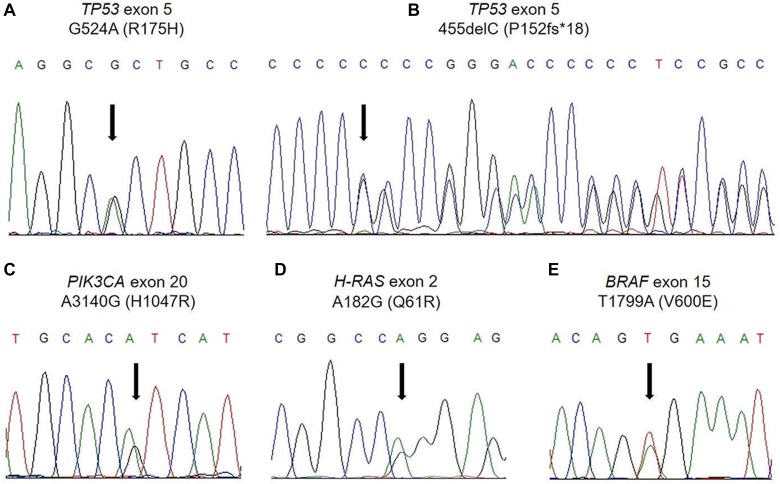
Examples of the direct DNA sequencing analysis (**A**) *TP53* missense mutation (R175H). (**B**) *TP53* frameshift mutation (P152fs^*^18). (**C**) *PIK3CA* mutation (H1047R). (**D**) *H-RAS* mutation (Q61R). (**E**) *BRAF* mutation (V600E).

*TP53* mutations were distributed throughout exons 4–10, and a subset of *TP53*-mutated cases simultaneously harbored missense, nonsense, and/or frameshift mutations in the same or different exons; 23 tumors (18% of the mutated tumors) carried more than one *TP53* mutation (Supplementary Table 1). The percentages of missense (Figure 1A), nonsense, and frameshift *TP53* mutations (Figure 1B) were 61%, 9.4%, and 27%, respectively, among the total mutations. The frameshift mutations (23 cases) included insertions (4 cases), deletions (19 cases) (Figure 1B), and in-frame mutations (2 cases). Consequently, the rates of each *TP53* status were as follows: wild-type, 32%; missense mutation, 42%; and truncating mutation, 26%. *PIK3CA* mutations were detected in either exons 9 (14 cases) or 20 (11 cases) (Figure 1C) (Supplementary Table 2). The majority of *H-RAS* mutations were identified in exon 2 (21/23 cases) (Figure 1D) (Supplementary Table 2). All of the *BRAF* mutations were represented as a V600E mutation (Figure 1E). In addition, an *AKT1* mutation was identified as an E17K mutation.

**Figure 2:**
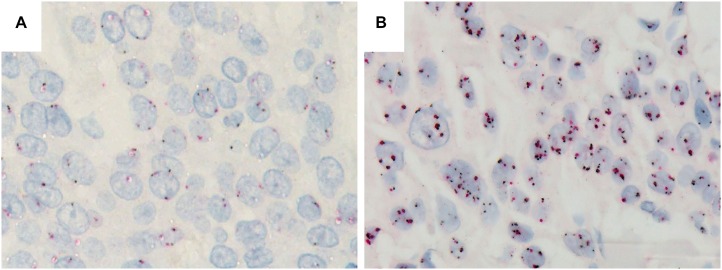
Dual color *in situ* hybridization for the *EGFR* gene (**A**) No abnormalities. (**B**) The presence of chromosome 7 polysomy but no *EGFR* gene amplification (*HER2* genes: black signal, CEN 17: red signal).

A dual color *in situ* hybridization (DISH) analysis revealed no *EGFR* gene amplification in any of the cases, whereas 14% (19/132 cases) of the cases demonstrated chromosome 7 polysomy (Figure 2).

**Figure 3:**
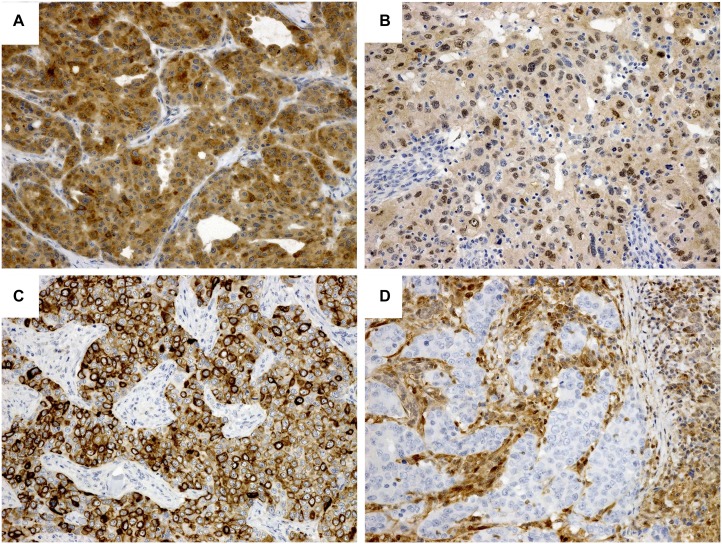
Immunohistochemical staining of the PI3K/Akt signaling pathway (**A**) PI3K. Diffuse cytoplasmic staining. (**B**) p-Akt. Half of the tumor cells show both nuclear and cytoplasmic staining. (**C**) p-mTOR. Many tumor cells are positive with cytoplasmic and membranous staining pattern. (**D**) PTEN. Expression loss. Note that the expression of stromal and adjacent salivary gland parenchymal cells is retained.

Positive immunoreactivity for PI3K and p-mTOR was exclusively observed in the cytoplasm, while p-Akt was sometimes detected in the nuclei and cytoplasm by immunostaining. The positive rate—in accordance with the sequential order of the signaling pathway axis—was as follows: PI3K, 34% (51/149 cases) (Figure 3A), p-Akt, 22% (32/147 cases), nuclear p-Akt, 14% (21/147 cases) (Figure 3B), and p-mTOR, 66% (98/149 cases) (Figure 3C). The loss of PTEN (Figure 3D) was observed in 49% of the cases (73/149 cases).

### Mutual relationship between the gene alterations and immunohistochemical findings

*PIK3CA* and *H-RAS* mutations, which were frequently co-mutated (*P* < 0.001) (Supplementary Table 2), had a mutually exclusive relationship with the HER2-positive status (*P* = 0.004 and < 0.001, respectively) (Table 2). In contrast, the HER2-positive cases commonly carried *TP53* mutations (*P* = 0.044). Meanwhile, *TP53* wild-type was associated with *H-RAS* mutations (*P* = 0.013). Cases harboring *H-RAS* mutations and chromosome 7 polysomy showed significantly lower and higher Ki-67 labeling index (LI) values, respectively (*P* = 0.001 and < 0.001, respectively). In addition, *TP53* and *PIK3CA* mutations tended to have higher and lower Ki-67 LI values, respectively (*P* = 0.057 and 0.069, respectively).

**Table 2:** Mutual correlation between gene alterations and immunohistochemical findings in salivary duct carcinomas

*­–*	*TP53*			*PIK3CA*			*H-RAS*			*BRAF*		
	WT	Mut	*P*	WT	Mut	*P*	WT	Mut	*P*	WT	Mut	*P*
HER2	­–	­–	­–	­–	­–	­–	­–	­–	­–	­–	­–	­–
Neg	27	41	0.044^*^	54	20	0.004^*^	53	22	< 0.001^*^	69	5	0.037^*^
Pos	13	44		58	5		64	1		62	0	
	­–	­–	­–	­–	­–	­–	­–	­–	­–	­–	­–	­–
*TP53*	­–	­–	­–	­–	­–	­–	­–	­­–	­–	­–	­–	­–
WT	­–	­–	­–	32	8	N.S.	28	12	0.013^*^	37	3	N.S.
Mut	­–	­–	­–	71	14		75	10		82	2	
	­–	­–	­–	­–	­–	­–	­–	­–	­–	­–	­–	­–
*PIK3CA*	­–	­–	­–	­–	­–	­–	­–	­–	­–	­–	­–	­–
WT	­–	­–	­–	­–	­–	­–	103	9	< 0.001^*^	108	3	N.S.
Mut	­–	­–	­–	­–	­–	­–	11	14		22	2	
	­–	­–	­–	­–	­–	­–	­–	­–	­–	­–	­–	­–
*H-RAS*	­–	­–	­–	­–	­–	­–	­–	­–	­–	­–	­–	­–
WT	­–	­–	­–	­–	­–	­–	­–	­–	­–	108	5	N.S.
Mut	­–	­–	­–	­–	­­­–	­–	­–	­–	­­­–	23	0	
	­–	­–	­–	­–	­–	­–	­–	­–	­–	­–	­–	­–
Ki-67 LI (%)^†^	37.4 ± 21.7	46.2 ± 23.8	N.S. (0.057)	46.0 ± 24.4	36.4 ± 21.4	N.S. (0.069)	46.9 ± 23.7	29.8 ± 20.0	0.001^*^	44.9 ± 24.3	32.0 ± 4.5	N.S.

^*^Statistically significant (*P* < 0.05); ^†^mean ± SD

Abbreviations: LI = labeling index; WT = wild type; Mut = mutation; Neg = negative; Pos = positive; N.S. = not significant.

With regard to the correlation between the *TP53*-mutation status and the p53-expression phenotype (Table 3) (Supplementary Table 1), *TP53* mutations were more frequently identified in p53-extreme negative/positive cases than in p53-non-extreme cases (*P* = 0.002). Moreover, significant correlations were found between p53-extreme negative and *TP53* truncating mutation and between p53-extreme positive and *TP53* missense mutations (*P* < 0.001).

**Table 3:** Correlation between p53-expression phenotype and TP53 status

p53-expression phenotype	n	*TP53* status		
		Wild type	Mutation	
			Missense	Truncating
Non-extreme	71	31^*^	40^*^	
			21	19
EN + EP	53	9^*^	44^*^	
EN	18	3	5^†^	10^†^
EP	35	6	26^†^	3^†^
Total	124	40	84	

^*^P = 0.002; ^†^*P* < 0.001

Abbreviations: EN = extreme negative; EP = extreme positive.

The presence of chromosome 7 polysomy tended (with marginal significance [*P* = 0.065]) to be correlated with EGFR overexpression.

The interrelationship between the mutation and expression of molecules related to the PI3K/Akt signaling pathway is shown in Table 4. PTEN loss was found to be connected to PI3K negativity and nuclear p-Akt positivity (*P* = 0.006 and 0.007, respectively). Furthermore, both the overall and nuclear p-Akt expression were positively correlated with p-mTOR expression (*P* = 0.032 and 0.034, respectively). On the other hand, the *PIK3CA* status was not associated with the expression of PI3K/Akt signaling pathway proteins.

**Table 4:** Interrelation between the mutation and expression of molecules associated with the PI3K/Akt signaling pathway in salivary duct carcinomas

	PI3K			PTEN			p-Akt			p-Akt (N)			mTOR		
	Neg	Pos	*P*	Loss	Intact	*P*	Neg	Pos	*P*	Neg	Pos	*P*	Neg	Pos	*P*
*PIK3CA*	–	–	–	–	–	–	–	–	–	–	–	–	–	–	–
WT	72	39	N.S.	58	53	N.S.	87	23	N.S.	96	14	N.S.	39	72	N.S.
Mut	17	8		9	16		18	7		19	6		6	19	
	–	–	–	–	–	–	–	–	–	–	–	–	–	–	–
PI3K	–	–	–	–	–	–	–	–	–	–	–	–	–	–	–
Neg	–	–	–	56	42	0.006^*^	78	19	N.S.	86	11	N.S.	36	62	N.S.
Pos	–	–	–	17	34		37	13		40	10		15	36	
	–	–	–	–	–	–	–	–	–	–	–	–	–	–	–
PTEN	–	–	–	–	–	–	–	–	–	–	–	–	–	–	–
Loss	–	–	–	–	–	–	52	20	N.S. (0.084)	56	16	0.007^*^	30	43	N.S. (0.083)
Intact	–	–	–	–	–	–	63	12		70	5		21	55	
	–	–	–	–	–	–	–	–	–	–	–	–	–	–	–
p-Akt	–	–	–	–	–	–	–	–	–	–	–	–	–	–	–
Neg	–	–	–	–	–	–	–	–	–	–	–	–	45	70	0.032^*^
Pos	–	–	–	–	–	–	–	–	–	–	–		6	26	
	–	–	–	–	–	–	–	–	–	–	–	–	–	–	–
p-Akt (N)	–	–	–	–	–	–	–	–	–	–	–	–	–	–	–
Neg	–	–	–	–	–	–	–	–	–	–	–	–	48	78	0.034^*^
Pos	–	–	–	–	–	–	–	–	–	–	–	–	3	18	

^*^Statistically significant (*P* < 0.05) Abbreviations: p-Akt (N) = p-Akt nuclear expression; WT = wild type; Mut = mutation; Neg = negative; Pos = positive; N.S. = not significant.

**Figure 4:**
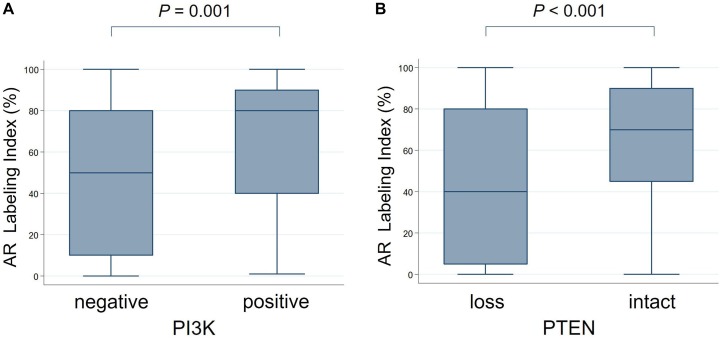
The comparison of androgen receptor labeling index (AR LI) with PI3K/PTEN expression PI3K negativity (**A**) and PTEN loss (**B**) are significantly associated with lower AR LI (PI3K: mean [%] ± SD, 47.6 ± 34.5 versus 66.2 ± 29.6; PTEN: 42.7 ± 34.8 versus 64.7 ± 29.6).

As reported previously, AR immunoreactivity was found in 144 of 150 cases (96%) [19]. Besides, PI3K (Figure 4A) and p-mTOR negativity, and the loss of PTEN (Figure 4B) were associated with a lower AR LI (*P* = 0.001, 0.098 [marginal significance], and < 0.001, respectively).

### Correlation between the molecular profile and clinicopathologic factors

The significant clinicopathological correlations included the presence of *PIK3CA* gene mutations with higher age (*P* = 0.025), PI3K and p-mTOR positivity with an early N classification (*P* = 0.039 and 0.028, respectively), and the loss of PTEN with an advanced T classification (*P* = 0.009).

Of note, the *PIK3CA* and *H-RAS* status showed significant differences according to the histological origin of SDC (Table 5). *PIK3CA* and *H-RAS* mutations were more commonly identified in *de novo* SDC than in SDC ex-PA (*P* < 0.001 for each). Similarly, the frequency of simultaneous *PIK3CA* and *H-RAS* mutations was also higher in *de novo* SDCs (*P* < 0.001). Even though the incidence was low, *BRAF* mutations were only found in *de novo* SDC. *AKT1* mutations were identified in one case each of *de novo* SDC and SDC ex-PA. The histological origin was not significantly associated with the *TP53* mutation status, chromosome 7 polysomy, or the expression of PI3K, p-Akt, p-mTOR, and PTEN.

**Table 5:** Association between the histologic origin and therapeutically-relevant genetic alterations in salivary duct carcinomas

Histologic origin	*TP53*			*PIK3CA*			*H-RAS*			*BRAF*			HER2^*^ (*ERBB2*^†^)		
	WT	Mut	*P*	WT	Mut	*P*	WT	Mut	*P*	WT	Mut	*P*	Neg	Pos	*P*
Current study															
*De novo*	20	29	N.S.	35	18	< 0.001^‡^	35	19	< 0.001^‡^	46	5	0.004^‡^	43	14	< 0.001^‡^
Ex-PA	19	52		72	7		77	4		80	0		35	54	
Griffith et al. (2013) (Ref. 18)															
*De novo*	N.A.			14	5	N.S.	N.A.			N.A.			N.A.		
Ex-PA				12	2										
Grünewald et al. (2016) (Ref. 14)															
*De novo*	14	18	N.S.	N.A.			N.A.			N.A.			N.A.		
Ex-PA	4	7													
Chiosea et al. (2016) (Ref. 13)															
*De novo*	7	1	0.033^‡^	2	6	0.010^‡^	3	5	0.029^‡^	8	0	N.S.	8	0	0.005^‡^
Ex-PA	14	17		23	8		24	7		29	2		14	17	

^*^HER2 status was assessed by a combination of immunohistochemistry and FISH analysis in accordance with the 2013 ASCO/CAP guideline. (Data from our previous report [Ref. 19])

^†^*ERBB2* positive was defined as > 8 genes gain by next-generation sequencing (Ref. 13).

^‡^Statistically significant (*P* < 0.05)

Abbreviations: PA = pleomorphic adenoma; WT = wild type; Mut = mutation; Neg = negative; Pos = positive; N.S. = not significant; N.A. = not available.

### Prognostic impact

The data of the univariate and multivariate Cox proportional hazard analyses of the molecular and immunohistochemical variables associated with survival are summarized in Table 6. The univariate analysis revealed that patients with *TP53* mutated SDCs showed poorer PFS in comparison to those with carried wild-type *TP53* (*P* = 0.017); however, the difference was not statistically significant in the multivariate analysis. Furthermore, the presence of a *TP53* truncating mutation was a significant independent predictor of poor PFS in both the univariate and multivariate analyses (*P* = 0.008 and 0.004, respectively). While, p-Akt-positive expression was associated with a favorable clinical outcome indicated by better OS and PFS in a univariate analysis (*P* = 0.017 and 0.023, respectively); these associations showed marginal trend in the multivariate analysis (*P* = 0.064 and 0.106, respectively).

**Table 6:** Adjusted by age, sex, primary site, TNM clasification, definitive treatment, and histologic origin

Molecules		n	Overall survival						Progression-free survival					
			Univariate analysis			Multivariate analysis			Univariate analysis			Multivariate analysis		
			HR	95% CI	*P*	HR	95% CI	*P*	HR	95% CI	*P*	HR	95% CI	*P*
*TP53*	*WT*	40	1.00	-	-	1.00	-	-	1.00	-	-	1.00	-	-
	*Mut*	85	1.81	0.97-3.37	N.S. (0.062)	1.56	0.82-2.97	N.S.	1.82	1.11-2.99	0.017^*^	1.45	0.87-2.44	N.S.
	*WT*	40	1.00	-	-	1.00	-	-	1.00	-	-	1.00	-	-
	*Missense*	52	1.94	1.00-3.75	0.049^*^	1.45	0.73-2.85	N.S.	1.64	0.97-2.79	N.S. (0.067)	1.13	0.64-1.98	N.S.
	*Truncating*	33	1.60	0.76-3.37	N.S.	1.88	0.84-4.21	N.S.	2.19	1.23-3.90	0.008^*^	2.54	1.35-4.76	0.004^*^
*PIK3CA*	*WT*	112	1.00	-	-	1.00	-	-	1.00	-	-	1.00	-	-
	*Mut*	25	0.75	0.38-1.46	N.S.	0.63	0.29-1.36	N.S.	0.77	0.45-1.32	N.S.	0.71	0.39-1.28	N.S.
*H-RAS*	*WT*	117	1.00	-	-	1.00	-	-	1.00	-	-	1.00	-	-
	*Mut*	23	0.69	0.36-1.35	N.S.	0.77	0.35-1.67	N.S.	0.77	0.44-1.33	N.S.	1.03	0.54-1.94	N.S.
*BRAF*	*WT*	131	1.00	-	-	1.00	-	-	1.00	-	-	1.00	-	-
	*Mut*	5	0.37	0.05-2.65	N.S.	0.27	0.03-2.09	N.S.	0.40	0.10-1.62	N.S.	0.36	0.08-1.55	N.S.
Chromosome 7 polysomy	*Absent*	113	1.00	-	-	1.00	-	-	1.00	-	-	1.00	-	-
	*Present*	19	0.66	0.27-1.66	N.S.	0.37	0.14-0.96	0.040^*^	1.25	0.69-2.25	N.S.	0.90	0.49-1.65	N.S.
PI3K	*Neg*	98	1.00	-	-	1.00	-	-	1.00	-	-	1.00	-	-
	*Pos*	51	0.65	0.38-1.13	N.S.	0.74	0.41-1.36	N.S.	1.01	0.68-1.52	N.S.	1.26	0.80-1.98	N.S.
p-Akt	*Neg*	115	1.00	-	-	1.00	-	-	1.00	-	-	1.00	-	-
	*Pos*	32	0.44	0.22-0.86	0.017^*^	0.50	0.24-1.04	N.S. (0.064)	0.54	0.32-0.92	0.023^*^	0.62	0.35-1.11	N.S. (0.106)
p-mTOR	*Neg*	51	1.00	-	-	1.00	-	-	1.00	-	-	1.00	-	-
	*Pos*	98	0.96	0.59-1.55	N.S.	1.42	0.83-2.44	N.S.	0.79	0.53-1.17	N.S.	1.02	0.65-1.60	N.S.
PTEN	Loss	73	1.00	-	-	1.00	-	-	1.00	-	-	1.00	-	-
	*Intact*	76	1.08	0.67-1.74	N.S.	1.04	0.61-1.76	N.S.	0.98	0.67-1.44	N.S.	1.00	0.65-1.55	N.S.

^*^Statistically significant (*P* < 0.05)

Abbreviations: HR = hazard ratio; CI = confidence interval; WT = wild type; Mut = mutation; Neg = negative; Pos = positive; N.S. = not significant.

**Figure 5:**
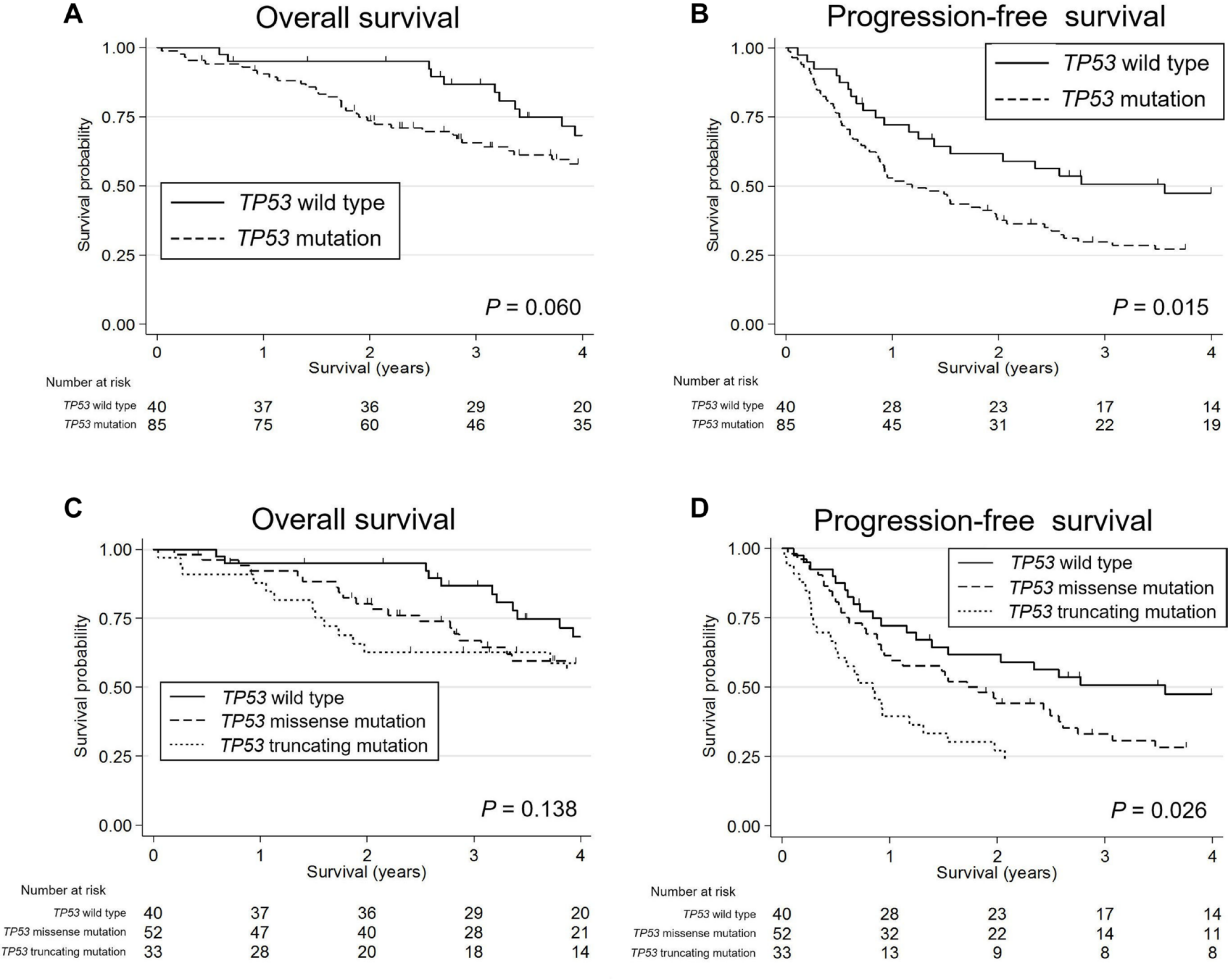
Kaplan-Meier survival curves of patients with salivary duct carcinoma stratified by their *TP53* status (**A**) *TP53* mutated patients tended to show poorer overall survival (OS) than *TP53* wild-type patients, with marginal significance. (**B**) The progression-free survival (PFS) of the *TP53* mutated patients was significantly worse than that of the *TP53* wild-type patients. (**C**) The OS of patients with *TP53* wild-type, missense mutations, and truncating mutations tended to decrease in this order. (**D**) The PFS of patients with *TP53* truncating mutations was significantly worst, followed in order by missense mutations and wild-type.

**Figure 6:**
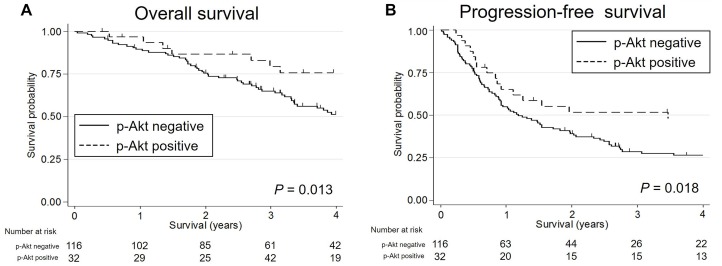
The Kaplan-Meier survival curves for patients with salivary duct carcinoma stratified by the p-Akt expression status Both overall survival (**A**) and progression-free survival (**B**) were significantly worse in p-Akt-negative patients than in p-Akt-positive patients.

Kaplan-Meier survival curves indicated that patients with *TP53* mutated SDC showed worse OS and PFS (*P* = 0.060 [marginal significance] and 0.015, respectively) than SDC patients with wild-type *TP53*; furthermore, *TP53* truncating mutations were significantly associated with poorest PFS (*P* = 0.026) (Figure 5). In the HER2-negative subgroup, a *TP53* mutation was an indicator of worse OS and PFS (*P* = 0.041 and 0.013, respectively). In contrast, p-Akt positivity was associated with higher OS and PFS (*P* = 0.013 and 0.018, respectively) (Figure 6). The other molecular and immunohistochemical factors that were examined showed no association with the survival rates.

## DISCUSSION

SDC has a very poor prognosis and the treatment options are limited [1–5]. We performed extensive molecular and immunohistochemical analyses of genes and biological markers either related to the driving tumorigenesis and/or relevant treatment targets in 151 cases of SDC. In patients with SDC, the most frequent gene alterations were observed in *TP53*, followed by *PIK3CA*, *H-RAS*, *BRAF*, and *AKT1*; this finding was in agreement with previous comprehensive genetic studies [9–17]. However, there is little in the way of convincing data in relation to the clinicopathological and prognostic implications of these molecular changes, mostly due to the rarity of SDC. Thus, the present study, which includes a large cohort of SDC patients, provides results that can more reliably interpret the associations between the mutation/expression status and clinicopathological features, including histogenesis and the clinical outcome. First, we found that *PIK3CA*/*H-RAS*/*BRAF* mutations were preferentially identified in *de novo* SDCs but not in SDCs ex-PA. These mutations and HER2 overexpression/amplification were mutually exclusive. Second, *TP53* mutations were common events in SDC, and the truncating form of the given gene was independently associated with a worse prognosis. Additionally, *TP53* mutations were frequently detected in cases with an extreme p53-negative/positive immunophenotype, and *TP53* missense and truncating mutations were associated with extreme p53 positivity and negativity, respectively. Finally, we demonstrated that at least one-fifth of SDC cases aberrantly expressed PI3K/Akt signaling pathway proteins, along with a mutual correlation with the expression profile according to the axis. Furthermore, we revealed that p-Akt positivity indicates a favorable clinical outcome. Moreover, PI3K negativity and PTEN loss were associated with low AR expression. We believe that in the future these data will be important in the implementation of personalized therapy for SDC.

SDC can occur as a *de novo* tumor, and can develop through malignant transformation from PA. It has been shown that the survival of patients with SDC ex-PA and those with *de novo* SDC does not differ to a statistically significant extent [20, 21]. Recently, studies analyzing small cohorts have shown that the underlying molecular mechanism and therapeutically-relevant genetic alterations in SDC might differ between *de novo* and ex-PA (Table 5) [13, 14, 18, 19]. However, conflicting results have been reported [13, 14, 18]. Chiosea et al. examined 39 cases and found that *PIK3CA* and *H-RAS* mutations were predominantly detected in *de novo* SDCs, whereas *TP53* mutations and *ERBB2* amplification were more commonly observed in SDCs ex-PA [13]. They investigated the presence of pre-existing PA based on a combination of histomorphology and the *PLAG1* and *HMGA2* rearrangement status. On the other hand, Griffith et al. failed to find an association between the *PIK3CA* status and the histologic origin in their analysis of 34 cases [18]. In the study of 43 cases, Grünewald et al. demonstrated that the *TP53* mutation rate was not influenced—to a statistically significant extent—by the histologic origin [14]. Our current analysis of much larger series of patients with SDC revealed hot spot mutations of *PIK3CA* and *H-RAS* in 18.2% and 16.2% of the cases, respectively, and showed the intimate relationship between the presence of *PIK3CA* mutations, frequently accompanied by simultaneous *H-RAS* mutations, and *de novo* occurrence. Although these findings confirm the data presented by Chiosea et al. [13], we identified cases of SDC ex-PA by a histomorphological evaluation using multi-step sections from the whole tumor. We found no significant association between the histological origin and the expression of PI3K, p-Akt, p-mTOR, and PTEN. Moreover, in agreement with the results presented by Grünewald et al., the *TP53* mutation rate did not differ according to the histologic origin [14]. Conversely, although the mutation rate was low, *BRAF* mutations (V600E)—one of the major therapeutic targets [22]—were only detected in *de novo* SDCs. Furthermore, our data showed that *PIK3CA/H-RAS/BRAF* mutations and HER2 positivity are mutually exclusive, and that SDCs ex-PA more frequently overexpressed HER family members, including HER2 (and/or *HER2* amplification), EGFR, and HER3 [19]. These findings indicate that *PIK3CA* and *H-RAS* mutations in *de novo* SDCs and the disruption of the HER family members in SDCs ex-PA may play crucial roles in their carcinogenesis. These insights should be considered when designing clinical trials and when planning potential targeted therapeutic strategies against SDCs based on the histological origin. Instead, consistent with previous studies, these molecular changes were not correlated with the patient outcome [8, 9, 18, 19]. The further clarification of the mechanisms associated with the development of SDCs is expected to lead to novel targeted therapies.

*TP53* is well known to be the most frequently mutated gene in human malignancies; mutations are found in at least 50% of human cancers [23–25]. In the current study, *TP53* mutations were found in 68% of the SDC cases, which is in line with previous reports [9, 13, 15, 16]. Pathogenic mutations result in the loss or abrogation of wild-type p53 activity and the dominant-negative effect on the remaining wild-type p53, in addition some p53 mutants often gain oncogenic functions to promote tumorigenesis and progression [26]. Given the broad role of p53 in malignancy, there is an intense focus on its potential as a therapeutic target [27, 28]. Furthermore, *TP53* mutations have been shown to be an independent marker of a poor prognosis in cancers of several organs [23–25]. Meanwhile, it should be noted that the prognostic significance of *TP53* mutations shows extreme variation according to the type of tumor [23–25]. In SDCs, it is not clear whether *TP53* mutation independently predicts a poor prognosis. Most recently, Grünewald et al. reported that *TP53* mutated SDCs tended to be associated with a worse OS; however, the result did not reach statistical significance due to the relatively small number of patients [14]. In the present study, *TP53* mutations were significantly associated with a poor PFS in a univariate analysis, but not in the multivariate analysis. A subgroup analysis using Kaplan-Meier survival curves showed that *TP53* mutations were associated with worse OS and PFS in HER2-negative patients. In general (not limited to SDCs) major *TP53* mutations are missense and cause single amino-acid changes at many different positions [23–25]. The mutations, however, are diverse in their type, sequence context, and structural impact—these differences influence the biological behavior of various cancers [23–25]. Missense substitutions, particularly certain “hot spots” mutations, have been shown to induce loss of DNA binding activity, transactivation capacity, and dominant-negative effects, as well as gain-of-function (GOF) properties, including altered cancer spectrum, deregulated metabolic pathways, increased metastasis and enhanced chemotherapy resistance [28–30]. Conversely, truncating *TP53* mutations, such as frameshift insertions and deletions and nonsense mutations, correspond to loss-of-function (LOF) *TP53* mutations and are thought to give rise to p53-null alleles [31–33]. In ovarian carcinomas, it has been suggested that distinguishing between GOF and LOF *TP53* mutations is clinically important as LOF mutations have been associated with worst prognosis in comparison to GOF mutations and wild-type [34, 35]. We found that in SDCs the rates of wild-type, missense mutation, and truncating mutation were 32%, 42%, and 26% of the cases, respectively. Thus, by dividing *TP53* status into 3 types (wild-type, missense mutations, and truncating mutations), we—for the first time—demonstrated that truncating mutations were a strongly independent adverse prognostic factor in SDC. Although mutations out of ‘‘hot spots’’ or large deletions/insertions might have been missed in our study, the molecular signatures of *TP53* are very useful for determining therapeutic strategies.

The p53 expression (which is determined by immunohistochemistry) is widely used as a surrogate marker of *TP53* mutation status; however, its reliability has not been established in SDCs. It has been generally accepted that wild-type p53 protein is relatively unstable and has a short half-life, which makes it undetectable by immunohistochemistry, whereas mutant p53 has a much longer half-life, and therefore accumulates in the nucleus creating a stable target for immunohistochemical detection [30]. However, the overexpression of wild-type p53 can sometimes occur due to impaired degradation under cellular stress [30]. On the other hand, truncating mutations invariably lead to the disruption of protein synthesis, and the biological consequences might differ from those of missense mutations [33]. In the present study of SDCs—as has been demostrated in breast [36] and ovarian carcinomas [37–39]—we proved that extreme p53 positivity and negativity was associated with *TP53* missense and truncating mutations, respectively; this possibility has been suggested previously [11, 14]. This could provide a reasonable explanation of why extreme p53 positivity/negativity was associated with a worse prognosis in SDCs, as was shown our recent study [19]. It should be noted that the absence of protein expression tends to confound the interpretation of immunostaining, leading to false-negative results, especially in the case of truncating mutations.

The PI3K/Akt pathway is central in the regulation of multiple cancer-relevant regulatory processes that affect cell survival, cell growth, and cell cycle progression [40–42]. PI3K and its downstream components (including Akt and mTOR) in turn cross-talk with a number of other pathways, thereby leading to a complex network of signals; the disruption of which may have dramatic consequences [40–42]. In addition, PTEN is a major negative regulator of the PI3K activity [40, 43, 44]. The deregulation of the PI3K/Akt pathway—including activation and somatic mutations—in this pathway is frequently found in various cancers; thus it represents an attractive target for therapy [40–42, 45, 46]. Aberrations in this pathway have also recently been implicated in salivary gland tumorigenesis [13, 18, 47–49]. We disclosed that the overexpression of PI3K, activated Akt (p-Akt), and activated mTOR (p-mTOR), and the loss of PTEN are common in SDC. Furthermore, PTEN loss was negatively and positively correlated to PI3K expression and its downstream p-Akt nuclear expression, respectively. Moreover, a positive correlation was found between the expression of p-Akt and the downstream expression of p-mTOR. These observations indicate that activation of the PI3K/Akt pathway through the axis plays a crucial role in the pathogenesis of a subset of SDCs. On the other hand, we have shown that *PIK3CA* mutations are not associated with the high expression of downstream-activated proteins; this finding is in line with other cancer studies [50, 51].

The correlation between the expression of the PI3K/Akt signaling pathway proteins and the clinical outcome of salivary gland carcinomas has not been fully clarified. To the best of our knowledge, this is the first study to report that p-Akt negativity is associated with an unfavorable prognosis in patients with SDC; however, similar findings have been described in relation to salivary adenoid cystic carcinoma [48] and “high-grade tumors,” without focusing on sub-entities such as SDC [47]. In the current study, the expression of PI3K, PTEN, and p-mTOR had no impact on the survival of SDC patients. Furthermore, we could not demonstrate any significant association between the *PIK3CA* status and the clinical outcome, even though wild-type *PIK3CA* tumors showed higher proliferation index values. Similar findings have been reported in breast cancer patients [51, 52].

It is well known that SDC demonstrates the high expression of AR, as was shown in our results [19]. Thus, AR has been the target of androgen deprivation therapy (ADT) for SDC in recent years [4, 5], similarly to prostate cancer [53]. The PI3K/Akt pathway is important in both androgen-sensitive and hormone-refractory prostate cancer, with functional cross-talk between the pathway and the AR activity involved in both the initiation and progression of prostate cancer [54–57]. Interestingly, we found that PI3K negativity and PTEN loss were significantly correlated with the lower expression of AR in SDC. This study may provide novel evidence demonstrating interaction between the PI3K/Akt and AR pathways in SDC, and may be the first step toward elucidating the mechanisms of ADT therapy.

In conclusion, the present study, which included the largest series of patients with SDC, confirms the molecular variability and heterogeneity of treatment-relevant targets in this tumor. Our data shows that the genetic mechanisms underlying carcinogenesis differ according to the histogenesis and highlight the importance of subclassifying SDC based on specific molecular abnormalities. Almost all gene mutations in *TP53*, *PIK3CA*, *H-RAS*, *BRAF*, and *AKT1* that were identified in this study were covered by the Catalogue of Somatic Mutations in Cancer (COSMIC), and most of these mutations are oncogenic, for which a functional analysis has already been performed [58–62]. However, an integrated molecular evaluation, including whole-exome sequencing, transcriptome and proteome analyses, and *in vitro* cell culture and patient-derived xenograft experiments, is warranted for gaining a greater understanding of the pathogenesis of this high-grade salivary gland carcinoma and the activity of targeted therapies. Besides, our analysis suggests that *TP53* truncating mutations, which are correlated with p53-extreme negative expression, is an adverse prognostic factor in SDC. Additionally, the activation of the PI3K/Akt pathway with interaction with AR, which shows some prognostic impact in SDC, implies that it has the potential to be a biomarker of tumor aggressiveness, which may be useful for selecting hormonal and targeted therapy. Further validation in clinical trials would be expected for the development of therapy selection and treatment strategy in patients with SDC.

## MATERIALS AND METHODS

### Patients and tissue specimens

The present multicenter collaborative retrospective study was approved by the Institutional Review Board of each facility.

In total, 151 SDC patients were diagnosed and treated at 7 institutions between 1992 and 2014. Patients who underwent anti-HER2 or anti-AR therapy as an initial treatment were excluded. These patients included cases that were previously reported by our collaborative facilities [2, 19, 63]. All of the cases in the central review system were correctly diagnosed by an expert pathologist (T.N.) based on strict histological criteria in accordance with the 2017 WHO Classification of Tumors [1]. In principle, multi-step sections from the whole tumor were subjected to a histological review. Cases were considered to be SDC ex-PA when a pre-existing PA component was histologically detected in the tumor, even if it was represented by a markedly hyalinized nodule. On the other hand, in cases where no PA component was identified in any of the sections examined, the tumor was diagnosed as *de novo* SDC. When only biopsy samples were available for examination, the histologic origin was categorized as “unknown” (*n* = 5). Tumor stage was determined according to the UICC TNM classification and staging system (2009, 7th edition). Clinical information was collected from the patients’ medical records by the person-in-charge in each facility.

### Mutational analysis

With microdissection methods, DNA of the carcinoma cell-rich part in each case was carefully extracted from the paraffin-embedded thin-sliced sections using a QIAmp DNA FFPE Tissue Kit (QIAGEN GmgH, Hilden, Germany), purified using QIAquick Spin Kit (QIAGEN GmgH), and subjected to a purity check using Nano Drop (Thermo Fisher Scientific, MA). A gene alteration analysis was performed for *TP53* (exons 4–10), *PIK3CA* (exons 9 and 20), *AKT1* (exon 2), *K-RAS* (exons 1-2), *N-RAS* (exons 1-2), *H-RAS* (exons 1-2), and *BRAF* (exon 15) by Sanger sequencing. The primer sequences are described in Supplementary Table 3.

### DISH

The *EGFR* gene copy number was assessed by a DISH analysis using Benchmark ULTRA (Ventana Medical Systems, CA); staining was performed according to the manufacturer's protocol (Roche, Basel Switzerland). Briefly, 20 tumor cells in which both EGFR signaling (black signal) and chromosome 7 centromere (CEN7) (red signal) were depicted and counted to calculate the ratio of the total number of EGFR signals to the total number of CEN7 signals. An EGFR/CEN7 ratio of ≥2.0 was considered to indicate *EGFR* gene amplification, whereas a mean CEN7 signal number of ≥3.0/cell was considered to indicate the presence of chromosome 7 polysomy.

### Immunohistochemistry

Immunohistochemical staining was performed for PI3K, p-Akt, p-mTOR, and PTEN using 4-μm-thick formalin-fixed, paraffin-embedded tissue sections. The antibodies used in the assay are shown in Supplementary Table 4. The cases were regarded as positive for PI3K, p-Akt, and p-mTOR when the percentage of cytoplasmic and/or nuclear staining cells was >10%. With regard to p-Akt, nuclear positivity was indicated as “p-Akt (N)”. The PTEN expression level was scored semiquantitatively, as described previously [64]. The normal surrounding epithelium served as an internal control. Tumor tissues were scored as follows: score 2, same staining intensity as that of the normal epithelium; score 1, staining intensity weaker than normal; score 0, no staining. We considered scores of 0 and 1 to indicate a loss of PTEN.

### Statistical analysis

The chi-squared test and Mann-Whitney *U* test were used to compare non-continuous and continuous variables, respectively. Univariate and multivariate Cox proportional hazards models and the Kaplan-Meier product-limit method were used to investigate the associations between the molecular/immunohistochemical results and OS and PFS. The potential confounders in the multivariate analysis included age, gender, TNM classification, primary site, first-line treatment, and histologic origin (*de novo* vs. SDC ex-PA). The strength of an association was determined by the hazard ratio and the 95% CI. The statistical analyses were performed using the STATA software program (version 13, StataCorp., College Station, TX). All of the tests were two-sided, and *P* values of < 0.05 were considered to indicate statistical significance.

## SUPPLEMENTARY MATERIALS FIGURES AND TABLES




