# Supplementary material for: Prognostic and histogenetic roles of gene alteration and the expression of key potentially actionable targets in salivary duct carcinomas
Source: Oncotarget. 2017 Dec 4;9(2):1852–67. doi: 10.18632/oncotarget.22927 (PMC5788604; doi:10.18632/oncotarget.22927)
Supplement: Supplementary file 1 [file oncotarget-09-1852-s001.pdf]

Prognostic and histogenetic roles of gene alteration and the expression of key potentially actionable targets in salivary duct carcinomas

SUPPLEMENTARY MATERIALS

Supplementary Table 1: Detail of the correlation between the p53-expression phenotype and *TP53* mutation. See_Supplementary_Table 1.

Supplementary Table 2: Detail of the correlation between the histologic origin and mutation of *PIK3CA*, *H-RAS*, *BRAF*, and *AKT1* in salivary duct carcinomas

Case No.	Histologic origin	*PIK3CA*	*H-RAS*	*BRAF*	*AKT1*
49	*De novo*	p.E542K (c.1624G > A) (exon 9)	p.G13R (c.37G > C) (exon 1)	WT	WT
74	*De novo*	p.E542K (c.1624G > A) (exon 9)	p.Q61R (c.182A > G) (exon 2)	WT	WT
77	*De novo*	p.E542K (c.1624G > A) (exon 9)	p.Q61R (c.182A > G) (exon 2)	WT	WT
12	*De novo*	p.E545G (c.1634A > G) (exon 9)	p.Q61R (c.182A > G) (exon 2)	WT	WT
23	*De novo*	p.E545K (c.1633G > A) (exon 9)	p.Q61R (c.182A > G) (exon 2)	WT	WT
133	*De novo*	p.E545K (c.1633G > A) (exon 9)	p.Q61R (c.182A > G) (exon 2)	WT	WT
55	*De novo*	p.H1047R (c.3140A > G) (exon 20)	p.Q61K (c.181C > A) (exon 2)	WT	WT
91	*De novo*	p.H1047L (c.3140A > T) (exon 20)	p.Q61K (c.181C > A) (exon 2)	WT	WT
95	*De novo*	p.H1047R (c.3140A > G) (exon 20)	p.Q61K (c.181C > A) (exon 2)	WT	WT
135	*De novo*	p.H1047R (c.3140A > G) (exon 20)	p.Q61K (c.181C > A) (exon 2)	WT	WT
93	*De novo*	p.H1047R (c.3140A > G) (exon 20)	p.Q61R (c.182A > G) (exon 2)	WT	WT
140	*De novo*	p.H1047R (c.3140A > G) (exon 20)	p.Q61R (c.182A > G) (exon 2)	WT	WT
63	*De novo*	p.E545K (c.1633G > A) (exon 9)	WT	p.V600E (c.1799T > A)	WT
113	*De novo*	p.H1047R (c.3140A > G) (exon 20)	WT	p.V600E (c.1799T > A)	WT
136	*De novo*	p.E545K (c.1633G > A) (exon 9)	WT	WT	WT
28	*De novo*	p.E545K (c.1633G > A) (exon 9)	WT	WT	WT
129	*De novo*	p.H1047R (c.3140A > G) (exon 20)	WT	WT	WT
126	*De novo*	p.W1051X (c.3153G > A) (exon 20)	WT	WT	WT
130	*De novo*	WT	p.G13R (c.37G > C) (exon 1)	WT	WT
8	*De novo*	WT	p.Q61K (c.181C > A) (exon 2)	WT	WT
138	*De novo*	WT	p.Q61K (c.181C > A) (exon 2)	WT	WT
10	*De novo*	WT	p.Q61R (c.182A > G) (exon 2)	WT	WT
38	*De novo*	WT	p.Q61R (c.182A > G) (exon 2)	WT	WT
79	*De novo*	WT	p.Q61L (c.182A > T) (exon 2)	WT	WT
100	*De novo*	WT	p.Q61R (c.182A > G) (exon 2)	WT	WT
94	*De novo*	WT	WT	p.V600E (c.1799T > A)	p.E17K (c.49G > A)
25	*De novo*	WT	WT	p.V600E (c.1799T > A)	WT
150	*De novo*	WT	WT	p.V600E (c.1799T > A)	WT
83	Ex-PA	p.E545K (c.1633G > A) (exon 9)	p.Q61R (c.182A > G) (exon 2)	WT	WT
144	Ex-PA	p.E545K (c.1633G > A) (exon 9)	p.Q61X (c.181C > T) (exon 2)	WT	WT
44	Ex-PA	p.E545K (c.1633G > A) (exon 9)	WT	WT	WT
88	Ex-PA	p.E545K (c.1633G > A) (exon 9)	WT	WT	WT
90	Ex-PA	p.E545K (c.1633G > A) (exon 9)	WT	WT	WT
20	Ex-PA	p.H1047R (c.3140A > G) (exon 20)	WT	WT	WT
82	Ex-PA	p.H1047R (c.3140A > G) (exon 20)	WT	WT	WT
34	Ex-PA	WT	p.Q61K (c.181C > A) (exon 2)	WT	WT
148	Ex-PA	WT	p.Q61R (c.182A > G) (exon 2)	WT	WT
116	Ex-PA	WT	WT	WT	p.E17K (c.49G > A)

Cf. In total, 151 SDC cases were histologically classified as follows: 57 *de novo* cases (38%), 89 ex PA cases (59%), 5 unknown cases (3%).

Abbreviations: PA = pleomorphic adenoma; WT = wild type.

Supplementary Table 3: PCR primers used for sanger sequencing

Gene	Direction	Sequence (5′ to 3′)
TP53 exon 4	Forward	TGCTCTTTTCACCCATCTAC
	Reverse	ATACGGCCAGGCATTGAAGT
TP53 exon 5	Forward	TTCCTCTTCCTGCAGTACTC
	Reverse	CAGCTGCTCACCATCGCT
TP53 exon 6	Forward	TCCCCAGGCCTCTGATTCC
	Reverse	TGACAACCACCCTTAACCC
TP53 exon 7	Forward	CAAGGCGCACTGGCCTCATC
	Reverse	CACAGCAGGCCAGTGTGCAG
TP53 exon 8	Forward	GATTTCCTTACTGCCTCTTGC
	Reverse	GTGAATCTGAGGCATAACTGC
TP53 exon 9	Forward	GACAAGAAGCGGTGGAG
	Reverse	CGGCATTTTGAGTGTTAGAC
TP53 exon 10	Forward	CAATTGTAACTTGAACCATC
	Reverse	GGATGAGAATGGAATCCTAT
PIK3CA exon 9	Forward	TGACAAAGAAGAGCTCAAAGC
	Reverse	TTAGCACTTACCTGTGACTCCA
PIK3CA exon 20	Forward	TGATGACATTGCATACATTCG
	Reverse	TGTGTGGAAGATCCAATCCA
AKT1 exon 2	Forward	AGGCACATCTGTCCTGGCAC
	Reverse	AAATCTGAATCCCGAGAGGCC
H-RAS exon 1	Forward	CAGGCCCCTGAGGAGCGATG
	Reverse	TTCGTCCACAAAATGGTTCT
H-RAS exon 2	Forward	TCCTGCAGGATTCCTACCGG
	Reverse	GGTTCACCTGTACTGGTGGA
K-RAS exon 1	Forward	GACTGAATATAAACTTGTGG
	Reverse	CTGTATCAAAGAATGGTCCT
K-RAS exon 2	Forward	GACTGTGTTTCTCCCTTCT
	Reverse	TGGCAAATACACAAAGAAAG
N-RAS exon 1	Forward	GACTGAGTACAAACTGGTGG
	Reverse	GGGCCTCACCTCTATGGTG
N-RAS exon 2	Forward	GGTGAAACCTGTTTGTTGGA
	Reverse	ATACACAGAGGAAGCCTTCG
BRAF exon 15	Forward	TCCTTTACTTACTACACCTCAGAT
	Reverse	AGTGGAAAAATAGCCTCAAT

Supplementary Table 4: Antibodies used

Antigen	Clone	Dilution	Source
PI3K (p110α)	C73F8	x 400	Cell Signaling Technology, MA
p-Akt (Ser473)	D9E	x 50	Cell Signaling Technology, MA
p-mTOR (Ser2448)	49F9	x 100	Cell Signaling Technology, MA
PTEN	D4.3	x 100	Cell Signaling Technology, MA
HER2	Polyclonal	x 400	Dako Cytomation, CA
EGFR	31G7	Ready-to-use	NICHIREI Biosciences Inc., Tokyo, JPN
AR	AR441	Ready-to-use	BIOCARE Medical LLC, CA
p53	DO-7[*]	x 200	Dako Cytomation, CA
Ki-67	MIB-1	x 100	Dako Cytomation, CA

[*]The epitope recognized by the antibody is located between the N-terminal amino acids 1 and 45 and possibly between aminoacids 37 and 45 of the human p53 protein. The antibody reacts with wild type and mutant type of the p53 protein.
