# Supplementary material for: Prognostic and histogenetic roles of gene alteration and the expression of key potentially actionable targets in salivary duct carcinomas
Source: Oncotarget. 2017 Dec 4;9(2):1852–67. doi: 10.18632/oncotarget.22927 (PMC5788604; doi:10.18632/oncotarget.22927)
Supplement: Supplementary file 2 [file oncotarget-09-1852-s002.docx]

| **Supplementary Table 1:** Detail of the correlation between p53-expression phenotype and *TP53* mutation in salivary duct carcinomas | | | |
| --- | --- | --- | --- |
| **Case No.** | **p53-expression phenotype** | **Mutation type** | **Mutation (exons 4-10)** |
| 57 | EN | M | p.P71L (c.212C>T) (exon 4) |
| 122 | EN | M | p.R181H (c.542G>A) (exon 5) |
| 55 | EN | M | p.R306Q(c.917G>A) (exon 8) |
| 144 | EN | M | p.E336V (c.1007A>T) (exon 10) |
| 47 | EN | M | p.S116F (c.347C>T) (exon 4), p.E287K(c.859G>A) (exon 8), p.P301Q(c.902C>A) (exon 8), p.R306Q(c.917G>A) (exon 8),  p.K319N (c.957G>C) (exon 9) |
| 66 | EP | M | p.R110P (c.329G>C) (exon 4) |
| 109 | EP | M | p.G154D (c.461G>A) (exon 5) |
| 56 | EP | M | p.R175H(c.524G>A) (exon 5) |
| 93 | EP | M | p.H179R(c.536A>G) (exon 5) |
| 50 | EP | M | p.Y205C (c.614A>G) (exon 6) |
| 36 | EP | M | p.H214R (c.641A>G) (exon 6) |
| 39 | EP | M | p.V216L (c.646G>T) (exon 6) |
| 24 | EP | M | p.Y220C (c.659A>G) (exon 6) |
| 37 | EP | M | p.G245S (c.733G>A) (exon 7) |
| 8 | EP | M | p.R248Q (c.743G>A) (exon 7) |
| 110 | EP | M | p.I255F (c.763A>T) (exon 7) |
| 7 | EP | M | p.E258K (c.772G>A) (exon 7) |
| 124 | EP | M | p.E258A (c.773A>C) (exon 7) |
| 40 | EP | M | p.R273C (c.817C>T) (exon 8) |
| 71 | EP | M | p.R273H (c.818G>A) (exon 8) |
| 75 | EP | M | p.P278S (c.832C>T) (exon 8) |
| 99 | EP | M | p.R280K (c.839G>A) (exon 8) |
| 105 | EP | M | p.D281N (c.841G>A) (exon 8) |
| 121 | EP | M | p.D281H (c.841G>C) (exon 8) |
| 112 | EP | M | p.R337L (c.1010G>T) (exon 10) |
| 18 | EP | M | p.P64L (c.191C>T) (exon 4), p.T118I (c.353C>T) (exon 4) |
| 5 | EP | M | p.P58S (c.172C>T) (exon 4), p.R273H (c.818G>A) (exon 8) |
| 46 | EP | M | p.T55I (c.164C>T) (exon 4), p.R282G (c.844C>G) (exon 8) |
| 103 | EP | M | p.P98L (c.293C>T) (exon 4), p.R273C (c.817C>T) (exon 8) |
| 69 | EP | M | p.S46F (c.137C>T) (exon 4), p.L348S (c.1043T>C) (exon 10) |
| 123 | EP | M | p.M243I;G244S (c.729_730GG>AA) (exon 7) |
| 82 | NE | M | p.M40V (c.118A>G) (exon 4) |
| 113 | NE | M | p.P58L (c.173C>T) (exon 4) |
| 135 | NE | M | p.A74V (c.221C>T) (exon 4) |
| 108 | NE | M | p.S90F (c.269C>T) (exon 4) |
| 26 | NE | M | p.S116F (c.347C>T) (exon 4) |
| 33 | NE | M | p.A161D (c.482C>A) (exon 5) |
| 98 | NE | M | p.I162N (c.485T>A) (exon 5) |
| 34 | NE | M | p.S166L (c.497C>T) (exon 5) |
| 132 | NE | M | p.Y236H (c.706T>C) (exon 7) |
| 44 | NE | M | p.M237I (c.711G>A) (exon 7) |
| 64 | NE | M | p.I251S(c.752T>G) (exon 7) |
| 54 | NE | M | p.G266V (c.797G>T) (exon 8) |
| 90 | NE | M | p.R280T (c.839G>C) (exon 8) |
| 73 | NE | M | p.E285K(c.853G>A) (exon 8) |
| 11 | NE | M | p.R342P (c.1025G>C) (exon 10) |
| 80 | NE | M | p.P75S (c.223C>T) (exon 4), p.P80L (c.239C>T) (exon 4) |
| 94 | NE | M | p.P64S (c.190C>T) (exon 4), p.E285G (c.854A>G) (exon 8) |
| 91 | NE | M | p.D281N(c.841G>A) (exon 8), p.T312I (c.935C>T) (exon 9) |
| 97 | NE | M | p.A161T (c.481G>A) (exon 5), p.L194F (c.580C>T) (exon 6),  p.F338L (c.1012T>C) (exon 10) |
| 130 | NE | M | p.D184N (c.550G>A) (exon 5), p.R202C (c.604C>T) (exon 6),  p.T256I (c.767C>T) (exon 7) |
| 129 | NE | M | p.P278L (c.833C>T) (exon 8), p.R283H (c.848G>A) (exon 8),  p.L289F (c.865C>T) (exon 8), p.S314F (c.941C>T) (exon 9),  p.P318S (c.952C>T) (exon 9), p.A364V (c.1091C>T) (exon 10) |
| 41 | EN | T | p.K101X (c.301A>T) (exon 4) |
| 136 | EN | T | p.L188fs*59 (c.561del T) (exon 6) |
| 139 | EN | T | p.R209fs*6 (c.625_626del AG) (exon 6) |
| 84 | EN | T | p.T211fs*5 (c.630_631ins A) (exon 6) |
| 85 | EN | T | p.E224fs*24 (c.669_670ins TT) (exon 6) |
| 146 | EN | T | p.R273fs*32 (c.818_820del GTG ins T) (exon 8) |
| 134 | EN | T | p.V73fs*50 (c.211del C) (exon 4), p.A86V (c.257C>T) (exon 4),  p.S96F (c.287C>T) (exon 4) |
| 20 | EN | T | p.D42N (c.124G>A) (exon 4), p.W91X (c.273G>A) (exon 4), p.M160fs*10 (c.477del C) (exon 5), p.R280K(c.839G>A) (exon 8) |
| 58 | EN | T | p.E62K (c.184G>A) (exon 4), p.Q104X (c.310C>T) (exon 4),  p.V122M (c.364G>A) (exon 4), p.P152fs*18 (c.455del C) (exon 5) |
| 68 | EN | T | p.P77S (c.229C>T) (exon 4), p.A86V (c.257C>T) (exon 4),  p.A88V (c.263C>T) (exon 4), p.Q192X (c.674C>T) (exon 6) |
| 141 | EP | T | in-frame mutation (c.732_735del CGGCins G) (exon 7) |
| 115 | EP | T | p.P322fs*23 (c.964del C) (exon 9) |
| 131 | EP | T | p.P151S (c.451C>T) (exon 5), p.S303X (c.908G>A) (exon 8) |
| 65 | NE | T | p.R110fs*13 (c.329del G) (exon 4) |
| 61 | NE | T | p.A138fs*32 (c.412del G) (exon 5) |
| 118 | NE | T | p.T150fs*16 (c.448_460del ACACCCCCGCCCG) (exon 5) |
| 89 | NE | T | in-frame mutation (c.598_613del ATTTGCGTGTGGAGT) (exon 6) |
| 116 | NE | T | p.R196X (c.586C>T) (exon 6) |
| 4 | NE | T | p.R213X (c.637C>T) (exon 6) |
| 27 | NE | T | p.L206fs*41 (c.617del T) (exon 6) |
| 13 | NE | T | p.R209fs*6 (c.625_626del AG) (exon 6) |
| 60 | NE | T | p.R209fs*6 (c.625_626del AG) (exon 6) |
| 107 | NE | T | p.R209fs*6 (c.625_626del AG) (exon 6) |
| 35 | NE | T | p.R283fs*23 (c.844_845ins G) (exon 8) |
| 87 | NE | T | p.Unknown (c.920-1_929del GCACTGCCCAA) (intron) |
| 53 | NE | T | p.Unknown (c.994-1delG) (intron) |
| 19 | NE | T | p.P64L (c.191C>T) (exon 4), p.R213X (c.637C>T) (exon 6) |
| 77 | NE | T | p.V73fs*50 (c.211del C) (exon 4), p.S106N (c.317G>A) (exon 4) |
| 92 | NE | T | p.P75fs*48 (c.224del C) (exon 4), P359L (1076C>T) (exon 10) |
| 117 | NE | T | p.Q100X (c.298C>T) (exon 4), p.P191fs*18 (c.569_570ins T) (exon 6), p.A353T (c.1057G>A) (exon 10) |
| 125 | NE | T | p.P36S (c.106C>T) (exon 4), p.Q52X (c.154C>T) (exon 4),  p.P87S (c.259C>T) (exon 4) |
| 12 | NE | T | p.P36S (c.106C>T) (exon 4), p.S116P (c.346T>C) (exon 4),  p.I254fs*5 (c.760_773del ATCATCACACTGGA) (exon 7),  p.R283H (c.848G>A) (exon 8) |
| Abbreviations: EP = extreme positive; EN = extreme negative; NE = non-extreme; M = missence; T = trancating. | | | |
|  |  |  |  |
